# BRCAShare: a video-based message can increase sharing of familial genetic test results

**DOI:** 10.1186/s13053-026-00344-z

**Published:** 2026-06-27

**Authors:** Katie Riefski, Amber Aeilts, Alexandra Spencer, Sue Friedman, Julia Cooper, Leigha Senter

**Affiliations:** 1 College of Medicine, The Ohio State University, Columbus, OH USA; 2 Division of Human Genetics, Department of Internal Medicine, The Ohio State University Comprehensive Cancer Center, Columbus, OH USA; 3 Facing Our Risk of Cancer Empowered (FORCE), Tampa, FL USA

**Keywords:** BRCA1, BRCA2, Cascade testing, Video, Family communication

## Abstract

**Background:**

Genetic testing for known familial pathogenic or likely pathogenic germline variants in cancer susceptibility genes is central to identifying relatives at heightened risk for cancer. However, current data suggest that uptake of this practice referred to as “cascade testing” remains suboptimal. Research demonstrates that healthcare providers can alleviate barriers to cascade testing by providing informational resources to facilitate patient-mediated sharing of their genetic test results with their at-risk relatives.

**Methods:**

We recruited 103 individuals with a pathogenic or likely pathogenic variant in *BRCA1*or *BRCA2 (BRCA1/2).* Participants were randomized to receive a family communication guide with or without a 2-minute video (BRCAShare) that describes the concept of a relative’s recent diagnosis of a harmful variant in *BRCA1/2*, meant to facilitate sharing of results with relatives. All participants were given surveys to assess reactions to and impact of BRCAShare across three domains: (1) participants’ sharing of genetic information and intent to share genetic information; (2) perceived susceptibility to and seriousness of *BRCA1/2* cancer related risks, benefits, or barriers to intrafamilial sharing of results and cascade testing; (3) the impact of family dynamic on sharing of genetic information.

**Results:**

The group given access to the BRCAShare video (guide + video) had significantly higher odds of reporting an intent to share and actual sharing with other family members compared to those who did not receive BRCAShare (guide only). Perceptions of cancer risk did not change significantly after viewing BRCAShare, however participants demonstrated strong understanding of risks and benefits of genetic testing at baseline. There was not a significant association between family dynamics and more intent to share.

**Conclusions:**

Our research suggests that this video message is a useful method for facilitating family sharing of *BRCA1*/*2* results.

## Introduction

Pathogenic and likely pathogenic variants (PV/LPV) in the *BRCA1* and *BRCA2* genes (*BRCA1/2*) pose a well-described hereditary risk for breast, ovarian, and other cancers [1, 2]. A crucial strategy to mitigate risk and improve health outcomes in families with this inherited cancer risk is cascade testing - the process by which germline testing is arranged for relatives of a person with a PV/LPV.

The United States Centers for Disease Control and Prevention and Office of Public Health Genomics (2014) named *BRCA1*/2 a Tier 1 Genomics Application, indicating that familial testing for *BRCA1*/2 has “significant potential for positive impact on public health based on available evidence-based guidelines and recommendations” [3]. However, despite the known benefit of cascade testing in this population, some studies estimate that pursuit of genetic testing by first- and second-degree relatives of probands with a *BRCA1/2* PV/LPV is 15% to 51% [4].

In most cancer genetics settings in the United States, patients who test positive for PV/LPVs are responsible for initiating the process of notifying their relatives of a familial PV/LPV and the opportunity to undergo cascade testing. The intrafamilial dissemination of genetic information is complex and variable, and there are several known barriers including the patient’s perceived burden of being the communicator of their genetic information, which includes managing the timing and content of the discussion, complex family dynamics, and fear or guilt over anticipated negative reactions of relatives [5, 6]. In contrast, one study determined that individuals who receive more information about their family’s mutation from their affected relatives are more likely to follow-up about the option of genetic testing, which underscores the importance of providing information to at-risk relatives [7].

We developed a two-minute animated video (BRCAshare) guided by the Health Belief Model (HBM) that conveys information about a relative testing positive for a PV/LPV in *BRCA1/2* [8]. Our previous work which surveyed unselected participants who viewed BRCAshare, concluded that viewers had improved knowledge and expressed that if they hypothetically received the BRCAshare video from one of their relatives, they intended to take action by either talking to a healthcare provider, talking to family members, or pursuing genetic testing [8, 9].

Here, we assess the usefulness of the same video in addition to a communication guide to actual patients with PV/LPVs in *BRCA1/2.*

## Methodology

### Procedure

We conducted a randomized trial to assess the impact of a two-minute animated video (BRCAShare) on intra-familial sharing of and intent to share genetic test results in individuals with *BRCA1/2* PV/LPVs. All participants received the FORCE (Facing Our Risk of Cancer Empowered) electronic communication guide. The cohort was then randomized 1:1 to receive BRCAShare (guide + video group) or not receive BRCAShare (guide only group). Of note, the REDCap randomization tool malfunctioned near the end of the enrollment period and as a result, 10 participants were assigned to the guide only group without actual randomization, which causes a small and unexpected assignment imbalance in randomization. Participants completed electronic surveys at baseline (T1), after receiving the communication guide (T2) and one week following randomization to guide only and guide + video groups (T3).

### FORCE family communication guide

The communication guide is an electronic PDF booklet developed by the advocacy group, FORCE. FORCE serves individuals and families facing hereditary breast, ovarian, pancreatic, prostate, colorectal, and endometrial cancers through education, support, advocacy and research efforts. The FORCE family communication guide and includes detailed information about issues to consider prior to sharing genetic information, with whom and what information to share, the process of sharing, and what to expect after sharing (https://www.facingourrisk.org/uploads/brochures/genes-between-us-en.pdf).

### BRCAShare video

Described previously in Aeilts et al. (2021), the BRCAShare video (http://www.brca-share.com) was developed and produced by study investigators at The Ohio State University in collaboration with Mills James Productions, who provided the artistry, animation, background music, and professional voiceover of the investigator-provided script. The script was written in second person and uses illustrated animation to describe the identification of a PV/LPV in *BRCA1/2* in a family member of the viewer. The content of the BRCAShare video was informed by the results of focus group interviews with *BRCA1/2* positive individuals and is guided by principles of HBM. The HBM proposes that an individual is more likely to engage in a behavior if they perceive greater risk (seriousness and susceptibility), perceive greater benefits than barriers to engagement in the behavior, and have been cued to action [9, 10]. The video includes information about cancer risks, examples of possible result-based health actions, focus-group identified barriers like concern about the cost of genetic testing, and a call to action.

### Participants

Eligible participants were adults between the ages of 18 to 80 who had a PV/LPV in *BRCA1/2* and had access to an internet-capable device. It was calculated that each group would contain a sample size of 50 to achieve 60.878% power to detect a difference between the group proportions of 0.2000. The proportion in the guide + video group is assumed to be 0.2000 under the null hypothesis and 0.4000 under the alternative hypothesis. The proportion in the guide only group is 0.2000. The test statistic used is the two-sided Z-Test with unpooled variance. The significance level of the test is 0.050. Participants were identified through clinical service providers in the Ohio State Division of Human Genetics and Mount Carmel Cancer Genetics Program in Columbus, Ohio. Providers introduced the study to their eligible clinical patients and requested verbal permission for study staff to contact them to discuss enrollment further. Additionally, a call for eligible participants was sent to the FORCE advocacy group mailing list, posted on their social media pages (Facebook and Twitter), and added to the research page on their website. When an eligible participant self-identified as interested, they were directed to contact OSU study personnel via email or telephone. Once contact was established, study personnel asked questions to assess eligibility and video communication preference. In each case, participant eligibility was verified and informed consent provided by all participants prior to inclusion in the study. All participants were offered a $5 gift card for each survey taken.

### Instrumentation

Upon enrollment, participants were prompted to complete the study baseline survey (T1). The baseline survey contained 58 items and collected demographic information, personal and family cancer history, and the date genetic test results were received. Additionally, the baseline survey measured the extent of prior genetic information sharing, family dynamics, and constructs of the HBM (seriousness, susceptibility, benefits, and barriers). HBM construct survey items were measured on a 5-point Likert scale from “strongly disagree” to “strongly agree” and included questions such as, “One or some of my relatives are likely to have a PV/LPV in *BRCA1/2*,” and “If my relatives have genetic testing, they are less likely to need cancer treatment someday.” These survey items were adapted from previously validated measures [10]. Intrafamilial relationship dynamics were measured using the Brief Family Relationships Scale (BFRS), which classifies a respondent’s impression of the quality of their family relationships [11]. The BFRS assesses three subscales (expressiveness, cohesion, and conflict). Possible scores on these subscales range from 16 to 70. Higher scores on the BFRS signify positive family dynamics (more communication, more support, and less conflict).

Upon completion of the baseline survey, participants were emailed or texted (based on their preference) a copy of the FORCE family communication guide. One week later, participants were sent a second survey (T2), which assessed reactions to, and perceptions of information presented in the family communication guide. The results of T2 are not described in this paper. Participants were then randomized via REDCap software to one of two study arms: guide only (*n* = 62) versus guide + video (*n* = 41). Following the completion of T2, those randomized to the video group were sent a link to the BRCAShare video. After randomization, all participants were sent the post-study survey (T3), which contained 40 items and measured genetic information-sharing intention and opinions. Intention was measured using a 5-point Likert scale from “unlikely” to “very likely” and assessed at three time points: since consenting to the study (intended to send in period of time between consent and follow up), within the next week, and within the next month. Additionally, the post-study survey (T3) re-evaluated the HBM constructs present in the baseline survey (T1) to assess for changes in response after viewing the communication guide and/or BRCAShare video.

### Data analysis

Baseline characteristics were described using counts and proportions, means (standard deviations), or medians (interquartile ranges), as appropriate. Descriptive statistics were also used to describe reactions to information presented in the BRCAShare video and to assess intention to share genetics information with family members.

Logistic regression models were used to compare intent to share genetic information and ease of sharing genetic information between the guide only group and the guide + video group. In this analysis, odds ratios reflected the odds of the video group answering “neutral,” “likely,” or “very likely” to share test result/BRCAShare video relative to the reference (guide only group). A priori, adjustment for time since receiving their *BRCA1/2* test result was considered to be a potentially important factor (on average, 3.7 years for the guide only group and 6.0 years for the guide + video group) related to past sharing of genetic information and future intent to share results with relatives, and it was included in adjusted models (15 participants had a missing test results date and were excluded from adjusted models). We collapse the Likert scale into a binary variable because of sparse counts for some of the outcomes, particularly in the higher endorsement categories. The intent was not to highlight respondents who did not report unwillingness to share.

Longitudinal linear mixed effects models were used to estimate the average differences in scores as well as the average differences in the change from baseline between randomized groups, while accounting for measurements within participants over time. These models included a random intercept for each participant and unstructured covariance structure. Changes in mean score from baseline to post-study survey for each randomized group were calculated, and differences in mean score in the post-study survey were compared between randomized groups (significance determined at *p* < 0.05). Confidence intervals and p-values are all two-sided and presented at the nominal level.

Lastly, we assessed the association between each of the three BFRS areas and four questions regarding sharing/intent to share genetic information using linear regression. Separate adjusted linear regression models each included a covariate for randomized group and time since receiving their *BRCA1/2* test result. Data management was conducted using SAS software, Version 9.4 (Copyright © 2023 SAS Institute Inc.). Analysis was performed in Stata (StataCorp. 2023. Stata Statistical Software: Release 16.1. College Station, TX: StataCorp LLC.).

## Results

In total, 172 individuals were screened and eligible for participation; 103 completed the baseline survey, and 92 participants completed all study surveys. Table 1 summarizes participant demographics, which reflects the homogeneity (predominantly female, white, highly educated) of participants across both cohorts.

**Table 1:** Demographics

Characteristics	Units		Guide Only *N =* 62	Guide + Video *N =* 41	Total *N =* 103
**Age**	Mean (SD)^a^		43.1 (13.4)	45.1 (13.3)	43.9 (13.3)
	Range^a^		22–69	27–77	22–77
**Race**	*N* (%)				
Hispanic/Latino			5 (8.1)	0 (0.0)	5 (4.9)
White (non-Hispanic)			53 (85.5)	37 (90.2)	90 (87.4)
Black (non-Hispanic)			2 (3.2)	0 (0.0)	2 (1.9)
Asian			1 (1.6)	3 (7.3)	4 (3.9)
Native American/ Alaska Native			1 (1.6)	0 (0.0)	1 (1.0)
Other			0 (0.0)	1 (2.4)	1 (1.0)
**Sex**	*N* (%)				
Male			3 (4.8)	2 (4.9)	5 (4.9)
Female			58 (93.5)	39 (95.1)	97 (94.2)
Prefer not to answer			1 (1.6)	0 (0.0)	1 (1.0)
**Highest degree of school**	*N* (%)				
Grade 12 or GED			1 (1.6)	1 (2.4)	2 (1.9)
College 1–3 years			9 (14.5)	3 (7.3)	12 (11.7)
College 4 years/more			52 (83.9)	36 (87.8)	88 (85.4)
Prefer not to answer			0 (0.0)	1 (2.4)	1 (1.0)
**Annual household income**	*N* (%)				
$10,000 – <$25,000			5 (8.1)	0 (0.0)	5 (4.9)
$25,000 – <$35,000			6 (9.7)	0 (0.0)	6 (5.8)
$35,500 – <$50,000			8 (12.9)	4 (9.8)	12 (11.7)
$50,000 – <$75,000			7 (11.3)	4 (9.8)	11 (10.7)
$75,000 – <$100,000			7 (11.3)	2 (4.9)	9 (8.7)
$100, 000 – <$200,000			17 (27.4)	17 (41.5)	34 (33.0)
> $200,000			6 (9.7)	5 (12.2)	11 (10.7)
Prefer not to answer			6 (9.7)	9 (22.0)	15 (14.6)
**Personal diagnosis of cancer**		*N* (%)			
Yes			20 (32.3)	16 (39.0)	36 (35.0)
No			42 (67.7)	23 (56.1)	65 (63.1)
I don’t recall			0 (0.0)	2 (4.8)	2 (2.0)

^a^Age is represented in years

Notably, over half (58%, *n* = 60) of total participants were not the first person in their family to have genetic testing for *BRCA1/2* and nearly all participants (99%, *n* = 102) reported they had already shared their *BRCA1/2* results with at least one close and/or distant family member. Still, 45% (*n* = 46) of participants reported having family members that they have not yet informed about their *BRCA1*/*2* PV/LPV.

### Familial sharing and intent to share

Participants were asked about sharing and intentions to share their genetic test results and/or the video, which heretofore will be referred to collectively as “genetic information.” The guide + video group reported more sharing of genetic information with family members (55%, *n* = 20) compared to the guide only group (38%, *n* = 21). Of the 45% that have uninformed relatives, approximately half (*n* = 20) reported that they are likely or very likely share this information with relatives in the future. Participants who viewed the BRCAShare video (guide + video group) were 3.35 times more likely to respond “yes” to sharing genetic information with other family members since enrolling in the study compared to participants randomized to the guide only group [aOR = 3.35, 95% CI = 1.28, 8.82; *p* = 0.014].

When asked the likelihood of sharing their result with at least one relative in the next week, participants randomized to the guide + video group had 3.19 times the odds of reporting “neutral,” “likely,” or “very likely” to share the video with at least one relative in the next week, compared to participants randomized to the guide only group (for the sharing of their test results) [aOR = 3.19, 95% CI = 1.28, 7.92; *p* = 0.008]. A similar proportion (around half) of participants in each randomized group cited intent to share their result and/or the video with at least one relative in the next month [aOR = 1.07, 95% CI = 0.46, 2.52; *p* = 0.876].

The post-study survey (T3) measured participant’s perceptions of the ease of sharing genetic information. Though not a significant difference [aOR = 1.73, 95% CI = 0.64, 4.62; *p* = 0.277], a higher proportion of participants in the guide + video group (77%, *n* = 27) reported that sharing would be easy compared to participants in the guide only group (68%, *n* = 38).

A significant difference was observed in response to the question “sharing my genetic information will take a lot of time” between the guide + video group (34%, *n =* 12) and the guide only group (57%, *n =* 32) [aOR = 2.56, 95% CI = 1.06, 6.19; *p* = 0.037]. Neither group considered technological skills to be a barrier to sharing genetic information.

### Health belief model constructs

Overall, at baseline most participants in both groups demonstrated understanding of risk, seriousness, benefits, and likelihood of barriers with no baseline difference between groups (Table 2). We observed a change after receipt of BRCAShare (T3 versus T1) in mean responses for two questions: “If I share this information with my relatives, I will be viewed as a good family member” and “If my relatives have genetic testing, they are less likely to need cancer treatment someday” (Table 3). Both items represent the HBM benefits construct and no significant differences were noted for other items within this construct.

**Table 2:** Health belief model responses at baseline

HBM Construct	Survey Variable	Group	SA/A	Neutral	D/SD	NA
			*N* (%)	*N* (%)	*N* (%)	*N* (%)
Risk	[The familial PV/LPV] will negatively impact my relative.	Guide Only Guide+Video	43 (69) 29 (71)	16 (26) 10 (24)	3 (4.8) 2 (4.8)	
	One/some of my relatives are likely to have the *BRCA* gene mutation that I have.	Guide Only Guide+Video	58 (94) 34 (83)	8 (6.5) 4 (9.8)	0 (0) 3 (7.3)	
	One/some of my relatives are likely to get hereditary cancer.	Guide Only Guide+Video	52 (84) 28 (68)	10 (16) 9 (22)	0 (0) 4 (9.8)	
	My relatives are more likely to get cancer than most other people.	Guide Only Guide+Video	50 (81) 30 (73)	10 (16) 6 (15)	2 (3.2) 5 (12)	
Benefits	If I share this information with my relatives, I will be doing a good thing for them.	Guide Only Guide+Video	56 (90) 37 (90)	4 (6.5) 4 (9.8)	1 (1.6) 0 (0)	1 (1.6)
	If I share this information with my relatives, I will be viewed as a good family member.	Guide Only Guide+Video	31 (50) 23 (56)	28 (45) 15 (37)	1 (1.6) 3 (7.3)	2 (3.2)
	My relatives could benefit from the information that comes from a genetic test.	Guide Only Guide+Video	60 (99) 39 (95)	1 (1.6) 1 (2.4)	1 (1.6) 1 (2.4)	
	My relatives could do things to lower their cancer risk if they have genetic testing.	Guide Only Guide+Video	56 (90) 39 (95)	5 (8.1) 1 (2.4)	1 (1.6) 1 (2.4)	
	If my relatives have genetic testing, they are less likely to need cancer treatment someday.	Guide Only Guide+Video	28 (45) 20 (49)	17 (27) 13 (32)	17 (27) 8 (20)	
	My relative will want to know about my genetic test result.	Guide Only Guide+Video	41 (66) 26 (63)	16 (26) 12 (29)	4 (6.5) 3 (7.3)	1 (1.6)
	My relative will be thankful to me if I tell them about my genetic test result.	Guide Only Guide+Video	32 (52) 24 (59)	25 (40) 11 (27)	4 (6.5) 4 (9.8)	1 (1.6) 2 (4.8)
Barriers	If I share this information with my relatives, it will cause them stress.	Guide Only Guide+Video	46 (74) 29 (71)	9 (15) 8 (20)	7 (11) 4 (9.8)	
	Genetic testing will probably not be covered by my relatives’ insurance company.	Guide Only Guide+Video	14 (23) 4 (9.8)	29 (47) 15 (37)	19 (31) 22 (54)	
			**Very Serious/ Serious**	**Neutral**	**Not Serious**	**NA**
Seriousness	If one/some of my relatives were to develop cancer, how serious would it be?	Guide Only Guide+Video	55 (89) 33 (81)	7 (11) 5 (12)	0 (0) 1 (2.4)	2 (4.8)

^a^Adjusted model includes a covariate for the time in months since the participant received their *BRCA1/2* test result. Of note, 15 participants had a missing test results date and were excluded from adjusted model

**Table 3:** Health belief model responses at post-study

Survey Question	Guide Only^a^ Mean (SE)	Guide + Video^b^ Mean (SE)	Difference (95% CI), *p*-value
**If I share this information with my relatives, I will be viewed as a good family member.**			
Pre score	3.69 (0.11)	3.80 (0.14)	
Post score	3.75 (0.12)	3.56 (0.14)	-0.19 (-0.55, 0.18), *p* = 0.312
Change from pre	0.06 (0.10)	-0.24 (0.12)	**-0.30 (-0.60**,** 0.00)**, ***p* = 0.053**
**If my relatives have genetic testing, they are less likely to need cancer treatment someday.**			
Pre score	3.21 (0.13)	3.37 (0.16)	
Post score	3.43 (0.13)	3.17 (0.17)	-0.26 (-0.67, 0.15), *p* = 0.221
Change from pre	0.22 (0.13)	-0.20 (0.16)	**-0.41 (-0.82**,** -0.01)**, ***p* = 0.044**

Scoring of Likert scales consists of “strongly disagree” = 1; “strongly agree” = 5

^a^ Guide only group: *N =* 62 completed baseline survey; *N =* 56 completed post-survey

^b^ Guide + video group: *N =* 41 completed baseline survey; *N =* 36 completed post-survey

### Brief family relationships scale

The baseline survey (T1) assessed participant family dynamics of expressiveness, cohesion, and conflict using the BFRS. On average, participants in this study scored relatively high on the BRFS, indicating positive family dynamics (Table 4). Ultimately, there was no significant difference in mean scores between randomized groups, and no association found between higher scores on the BFRS scales and increased sharing or intent to share genetic information.

**Table 4:** Family dynamics

Family Relationship Scale	Guide Only *N =* 62 mean (SD) [range]	Guide + Video *N =* 41 mean (SD) [range]	Total *N =* 103 mean (SD)
Cohesion Score	20.3 (4.4) {5.0, 25.0}	20.4 (4.0) {11.0, 25.0}	20.3 (4.2)
Expressive Score	11.0 (3.1) {3.0, 15.0}	11.2 (2.5) {5.0, 15.0}	11.1 (2.9)
Conflict Score	23.8 (4.4) {12.0, 30.0}	24.0 (4.2) {12.0, 30.0}	23.9 (4.3)

Potential scores on these subscales range from 5 to 25 for cohesion, 3–15 for expressiveness, and 6–30 for conflict

## Discussion

Considering the persistently low uptake of cascade testing for familial *BRCA1/2* PV/LPVs, new methods to conveniently and efficiently convey information about *BRCA1/2* to at-risk relatives are needed. Bearing in mind the prevalence of technology-usage today, digital messaging may be a helpful tool to facilitate the cascade testing process within families with *BRCA1/2* PV/LPVs. According to U.S. Census Bureau data, approximately 92% of Americans have access to a computer and 85% to the internet [12]. Our results show that those who received the BRCAShare video were significantly more likely to share information about their familial *BRCA1/2* PV/LPV than those without the video. Additionally, participants who received the video did not perceive the sharing of their results to be time-consuming, whereas the guide only group had significantly higher odds of reporting that sharing their test results would take a lot of time. Taken together, these results suggest that a digital message may ease some of the communication burden that has been previously cited in medical literature as a barrier to cascade testing [5]. There is a growing body of evidence that demonstrates that various types of digital communication tools including chatbots and online educational modules can be helpful in facilitating cascade testing within families. One study described a chatbot that was used by 100 individuals with various cancer gene PV/LPV in the gynecologic oncology setting. Of the 100 individuals, 98.3% of those with eligible relatives accepted the chatbot, and by 3-month follow up, 83.8% had shared it with at least one relative. Furthermore, among 96 relatives who were reached by three months, 51% had scheduled or completed a genetics appointment [13, 14]. This success was demonstrated in other chatbot studies, as well as web-based platforms, which involve varying degrees of resources for the proband and their at-risk relatives [15–23]. Our results contribute to this expanding collection of evidence.

Guiding the development of the interventional video and the measures utilized in this study was the HBM, which was initially described by Rosenbeck in 1966 and has since been modified and adapted to serve as the basis of many health-related behavioral theories. The HBM proposes that an individual is more likely to engage in a behavior if they perceive greater risk (severity and susceptibility), perceive greater benefits than barriers to engagement in the behavior, and have been cued to action. Because communication efforts to encourage cascade testing are generally focused on informing and increasing risk perceptions among at-risk relatives, as well as educating about the possible the benefits of testing, this is an appropriate framework for this study.

Lack of change in post-intervention HBM construct measures is perhaps not surprising given that the cohort as a whole entered the study with rich experience and understanding of *BRCA1/2* risks and cascade testing options. At baseline (before viewing the communication guide or video), over 80% of both groups agreed their relatives were likely to have the familial *BRCA1/2* PV/LPV; over 95% agreed that their relatives could benefit from the information that comes from a genetic test; and over 90% agreed that their relatives could do things to lower their cancer risk if they have genetic testing. While we observed a statistically different change in mean response for two HBM construct measures related to perceived benefits, the remaining five measures of benefits demonstrated no change.

We assessed the impact of family dynamic on familial communication about test results. Published data suggest that the relative closeness of family members contributes to the decision to do cascade testing [24]. In this study we expected higher scores on the BFRS to correlate to more intent to share test results or the video, but both groups demonstrated positive family dynamics at baseline. Both groups had a high mean expressive score (11.1), cohesion score (20.3), and conflict score (23.9). It is possible that the impact of family dynamic on genetics information sharing may be more illuminating in a varied study population.

The BRCAShare video is a quick, well received, evidence-based intervention to increase the sharing of genetic information within families. The video is intended to reduce barriers to familial communication, which were explored in this study. While actual sharing is just a proximal outcome to the intent to share, our data support that the BRCAShare video begins to address barriers to communication of genetic information. It is possible that, coupled with additional resources, BRCAShare can more deeply and permanently address barriers to family sharing of genetic information. Currently, the BRCAShare video is available online at www.brca-share.com.

## Limitations

In this study, we have illustrated the ability of the BRCAShare video to increase family communication about and sharing of *BRCA1/2* test results and availability of cascade testing. However, some study limitations are noted. We did not exclude participants if they had already engaged in familial communication about their results. Consequently, participants’ intent to share may have been influenced by their history of prior genetics information sharing and an existing familiarity with the cancer predisposition syndrome. The reactions to and impact of the BRCAShare video in families with a newly identified PV/LPV in *BRCA1/2* and limited history of prior intrafamilial sharing of genetics information, should be explored in future studies where the video is presented at the time of result disclosure prior to familial engagement.

The study is also limited by lack of participant diversity. A more racially, ethnically, and socioeconomically diverse study population could also yield different study outcomes.

## Data Availability

The datasets used and/or analysed during the current study are available from the corresponding author on reasonable request.

## Abbreviations

PV/LPV: Pathogenic and likely pathogenic variants
BRCA1/2: BRCA1 and BRCA2 genes
HBM: Health Belief Model
BRCAshare: A two–minute animated video guided by the HBM that conveys information about a relative testing positive for a PV/LPV in BRCA1/2
FORCE: Facing Our Risk of Cancer Empowered
T1: Study baseline survey
T2: Second survey
T3: Post–study survey
BFRS: Brief Family Relationships Scale
